# Formulation and Evaluation of Valproic Acid Microemulsions for Enhanced Transfollicular Delivery in Guinea Pig Skin

**DOI:** 10.1111/jocd.16685

**Published:** 2024-11-20

**Authors:** Anayatollah Salimi, Soroush Jafarian, Arghavan Salimi, Saeed Mohammad Soleymani

**Affiliations:** ^1^ Department of Pharmaceutics, Faculty of Pharmacy Ahvaz Jundishapur University of Medical Sciences Ahvaz Iran; ^2^ Nanotechnology Research Center Ahvaz Jundishapur University of Medical Sciences Ahvaz Iran; ^3^ Student Research Committee Ahvaz Jundishapur University of Medical Sciences Ahvaz Iran; ^4^ Clinical Research Development Centre, Imam Hossein Educational Hospital Shahid Beheshti University of Medical Sciences Tehran Iran; ^5^ Department of Clinical Pharmacy, School of Pharmacy Shahid Beheshti University of Medical Sciences Tehran Iran

**Keywords:** drug delivery system, microemulsion (ME), permeability, transfollicular, valproic acid

## Abstract

**Background:**

Valproic acid (VPA) is used to treat various neurological and psychiatric conditions. While oral VPA can cause hair loss, topical application has shown potential for hair regeneration. This study aimed to develop and evaluate microemulsion (ME) formulations of VPA for enhanced transfollicular delivery.

**Methods:**

VPA‐loaded MEs were prepared using oleic acid, Transcutol P, Tween 80, Labrasol, and Capryol 90. The MEs were characterized for physicochemical properties, stability, in vitro release, and ex vivo permeation through the hairy abdominal and nonhairy ear skin of guinea pigs.

**Results:**

Eight stable ME formulations were developed with droplet sizes ranging from 10 to 24 nm, pH 4.6 to 5.2, and viscosity 77 to 85 cps. In vitro release studies showed controlled release profiles over 24 h. Permeation studies revealed enhanced drug delivery through both follicular and nonfollicular pathways compared with aqueous VPA solution. Formulations with higher surfactant/cosurfactant ratios showed increased permeation through the follicular pathway.

**Conclusion:**

The ME formulations significantly enhanced VPA penetration into both epidermal and follicular pathways compared with aqueous solution. The composition of the MEs, particularly the oil content, water content, and surfactant/cosurfactant ratio, played a crucial role in determining the physicochemical properties and skin permeation parameters of VPA.

## 1 Introduction

**FIGURE 1:**
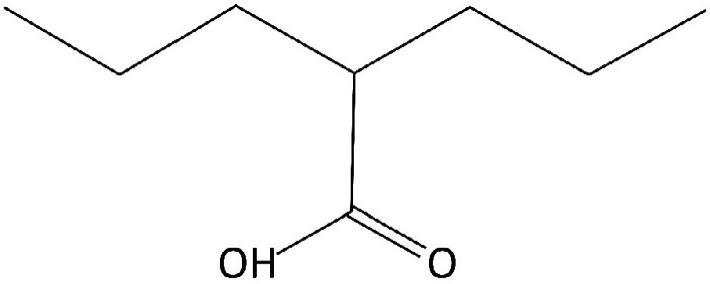
Chemical structure of valproic acid.

Valproic acid (VPA) is an eight‐carbon molecule with a pentatonic acid central structure and a propyl group attached to the second carbon. This medicine has a pKa of 4.8 and is a weak organic acid, and despite its good lipophilicity (log*P* = 2.75), it needs a carrier protein for its cellular uptake (Figure 1; [1]). VPA is commonly used to treat various seizure disorders, bipolar disorder, migraines, and other conditions off‐label [2, 3]. As a histone deacetylase inhibitor, it modifies chromatin structure and gene expression. VPA also affects ERK and Wnt‐beta‐catenin signaling pathways [4, 5]. It inhibits cellular sodium influx by blocking voltage‐dependent sodium channels and increases chloride influx through a GABA‐mimetic effect. Additionally, VPA reduces GABA release, which dampens neuronal excitation caused by glutamate receptors [6].

Several case studies have documented VPA‐induced hair loss. Patients receiving sodium valproate for psychiatric conditions experienced alopecia, with patients on 800 mg/day long‐term treatment becoming noncompliant due to hair loss. In epilepsy patients, chronic valproate therapy increased hair loss, which improved 84 days after discontinuing the drug [7].

VPA may cause hair loss through telogen effluvium and by increasing susceptibility to alopecia. Oral VPA administration has been linked to deficiencies in trace elements such as copper, zinc, and magnesium, as well as the inactivation of metallic enzymes involved in keratinization and hair growth [8].

Interestingly, while oral VPA intake causes hair loss, topical application has shown promise in hair regeneration. A South Korean study using murine models and human dermal papilla cells examined topical VPA's effect on androgenic alopecia. Male C3H mice treated with topical VPA experienced hair regrowth. Comparing VPA and minoxidil effects on human dermal papilla cells revealed that VPA significantly increased beta‐catenin, alkaline phosphatase, and BMP4 expression, while minoxidil did not [9].

VPA inhibits glycogen synthase kinase 3ß and activates the Wnt/βcatenin pathway, associated with hair regeneration and anagen induction [9]. Preclinical rat studies showed that higher VPA doses increased acetylation and activation of histone‐3 and PI3K pathway proteins. Lower split doses of VPA demonstrated cytoprotective abilities [10].

Microemulsions (MEs) are isotropic, thermodynamically stable dispersions containing oil, surfactant, cosurfactant, and an aqueous phase [11]. They are effective carriers for transdermal drug delivery, improving drug solubility and creating a high concentration gradient toward the skin. MEs offer several advantages, including simple preparation, drug solubilization capacity, and permeation enhancement by altering the stratum corneum barrier. Some ME components, such as oils and surfactants, may act as enhancers by disrupting the stratum corneum structure. MEs can improve the solubility of both hydrophilic and lipophilic drugs, potentially increasing the drug's thermodynamic activity in the skin and enhancing penetration [12, 13, 14, 15].

Against this background, this research aimed to develop a formulation for the transfollicular permeability of VPA from guinea pig hair.

## 2 Materials and Methods

### 2.1 Materials

VPA was obtained from Darou Pakhsh Pharmaceutical (Tehran, Iran). Caprylocaproyl Polyoxyl‐8 glycerides (Labrasol), Propylene glycol monocaprylate (Capryol 90), and Diethylene glycol mono ethyl ether (Transcutol P) were provided by Gattefosse (Saint‐Priest, France). Oleic acid and tween 80 were acquired from Merck (Germany). The Minitab 17 software was used for experimental design and statistical analysis.

### 2.2 Animal Studies

The Ethics Committee of Jundishapur University of Medical Sciences (IR.AJUMS.ABHC.REC.1399.004) on 03.03.2020 provided ethics approval for this study. Adult male guinea pigs weighing 300–500 g were obtained from Razi Institute, Karaj, Iran. The animals were provided with a standard diet and water. All procedures followed the principles for the care and use of laboratory animals, as outlined by the National Academy of Sciences and published by the National Institutes of Health (US Department of Health & Human Services, Office of Laboratory Animal Welfare).

### 2.3 The Solubility of Valproic Acid

VPA solubility was determined in oleic acid, oleic acid + Transcutol P (10:1), and oleic acid + surfactants (Tween 80, Labrasol), and cosurfactant (Capryol 90). An excess VPA was dissolved in 5 mL of each solution or mixture. Samples were mechanically mixed for 72 h at 25°C ± 0.5°C. After equilibration, the clear supernatants were filtered through a polytetrafluoroethylene membrane filter (0.45 μm). The filtrates were then analyzed using a UV spectrophotometer at 214 nm [16].

### 2.4 ME Preparation Based on Phase Diagram Construction

A previously prepared phase diagram by Salimi et al. was used [17]. Microemulsion properties can be influenced by the surfactant/cosurfactant ratio (S/C), water percentage (%w), and oil percentage (% oil). A full factorial design was employed to study these three variables at two levels. Eight MEs were prepared with 5% and 10% oil, 20%–30% water, 4:1 and 6:1 S/C ratios, and 8.3% VPA (Table 1).

**TABLE 1:** Composition of selected VPA microemulsions.

Formulation no.	Factorial	S/C	% Oil	% (S/C)	% Water
ME‐1	+++	6:1	10	60	30
ME‐2	++−	6:1	10	70	20
ME‐3	+−+	6:1	5	65	30
ME‐4	+−−	6:1	5	75	20
ME‐5	−−−	4:1	5	75	20
ME‐6	−−+	4:1	5	65	30
ME‐7	−+−	4:1	10	70	20
ME‐8	−++	4:1	10	60	30

The oil phase containing 8.3% VPA was combined with the S + C mixture. Double‐distilled water was then added dropwise to this mixture while stirring at room temperature until a uniform mixture formed [18].

### 2.5 Droplet Size Measurement

Particle size was measured using SCATTER SCOPE 1 QUIDIX (South Korea), which employs photon correlation spectroscopy. Measurements were taken at 25°C with a size range of 1–7000 nm.

### 2.6 Viscosity Determination

The rheological behavior of MEs was assessed using a Brookfield viscometer (DV‐II + Pro Brookfield, USA) at 25°C ± 1°C with spindle 34 at 50 rpm. Each 10 mL sample was used for viscosity measurements [19].

### 2.7 pH Measurement

The pH values of the MEs were measured at 25°C using a calibrated glass electrode (Mettler Toledo seven easy, Switzerland).

### 2.8 Physical Stability Studies

The International Conference on Harmonization of Technical Requirements for Medicines for Human Use (ICH) with the World Health Organization (WHO) published a set of sustainability guidelines in the early 2000s, which became known as the ICH guidelines [20]. Following ICH guidelines, MEs were stored at different temperatures (4°C, 25°C, 37°C and 75% ± 5% RH) for 6 months and visually inspected for phase separation and precipitation. Additionally, samples underwent centrifugation at 15 000 rpm for 30 min at 25°C ± 1°C (MPV‐350R, Poland) and were visually examined [18].

### 2.9 Drug Release Experiment

Custom‐designed static Franz diffusion cells (area 4.906 cm^2^) with a cellulose membrane (3000–4000 KD cutoff) were used to determine the release rate of VPA from various MEs.

Two grams of VPA‐loaded ME was carefully weighed and placed on the membrane. Phosphate buffer solution (PBS) at pH 7.4 was the receptor medium. The receptor fluid was stirred at 200 rpm using magnetic bars. At specific time intervals (0.5, 1, 2, 3, 4, 5, 6, 7, 8, and 24 h), 2 mL samples was taken from the receptor compartment, and the released drug was measured at 214 nm. The cumulative percentage of the released drug was plotted against time, and release data were fitted to zero‐order, first‐order, and Higuchi kinetic models. The model with the highest correlation coefficient (*r* ^2^) was selected [11].

### 2.10 Permeability Experiments

To examine VPA ME permeation through follicular and epidermal pathways, hairy abdominal skin and nonhairy pig ear skin were used, respectively [19]. Vertical diffusion cells (effective diffusion area approximately 4.906–0.3846 cm^2^) were used for in vitro permeation studies through hairy and nonhairy guinea pig skin. The receptor compartments contained 35 mL and 10 mL of PBS (pH 7.4), respectively. Prehydrated skin samples were mounted between the donor and receptor compartments, with the stratum corneum facing the donor medium. The donor phases were filled with VPA‐loaded MEs (3 g and 0.5 g). The diffusion cells were placed in a water bath at 37°C ± 0.5°C, and the receptor phase was stirred at 200 rpm. At each time point (0.5, 1, 2, 3, 4, 5, 6, 7, 8, and 24 h), 2 mL and 0.5 mL samples were collected from the receptor mediums and replaced with fresh PBS. A UV spectrophotometer measured the permeated VPA in the samples at 214 nm. An aqueous VPA solution with the same concentration as the MEs served as a control. Results were plotted as cumulative permeated drug percentage versus time. From these plots, the apparent permeability coefficient (Equation 1) and steady‐state permeation flux (*J*_ss_) (Equation 2) were calculated [21].

(1) $$P_{\mathrm{app}}=\frac{\mathrm{dQ}}{\mathrm{dt}}\times \frac{1}{A.C_{0}}$$

(2) $$J_{\mathrm{ss}}=C_{0}\times P_{\mathrm{app}}$$

where dQ/dt is the steady‐state appearance rate on the acceptor side of the skin, A is the skin area, and *C_0_* is the initial drug concentration in the donor phase.

### 2.11 Data Analysis

The steady‐state permeation rate (*J*_ss_) of VPA through the unit skin area was calculated from the linear portion of the permeation curve slope. One‐way analysis of variance (ANOVA) was used to identify significant differences. A *p* value < 0.05 was considered statistically significant, with 95% confidence intervals [22]. All statistical analysis was performed using the MINITAB software (version 17.0) [23].

## 3 Results

### 3.1 VPA Solubility

Table 2 presents the solubility data for VPA.

**TABLE 2:** VPA solubility in oils, surfactants, and cosurfactants (mean ± SD, *n* = 3).

Phase type	Excipient	Solubility (mg/mL)
Oil	Oleic acid	78.0 ± 3.0
	Transcutol‐P	85.5 ± 2.0
	Oleic acid + Transcutol‐P	81.5 ± 1.5
Surfactant	Tween 80	99.0 ± 1.0
	Labrasol	88.0 ± 3.0
Co‐surfactant	Capryol 90	61.5 ± 1.0

### 3.2 Phase Behavior Studies

**FIGURE 2:**
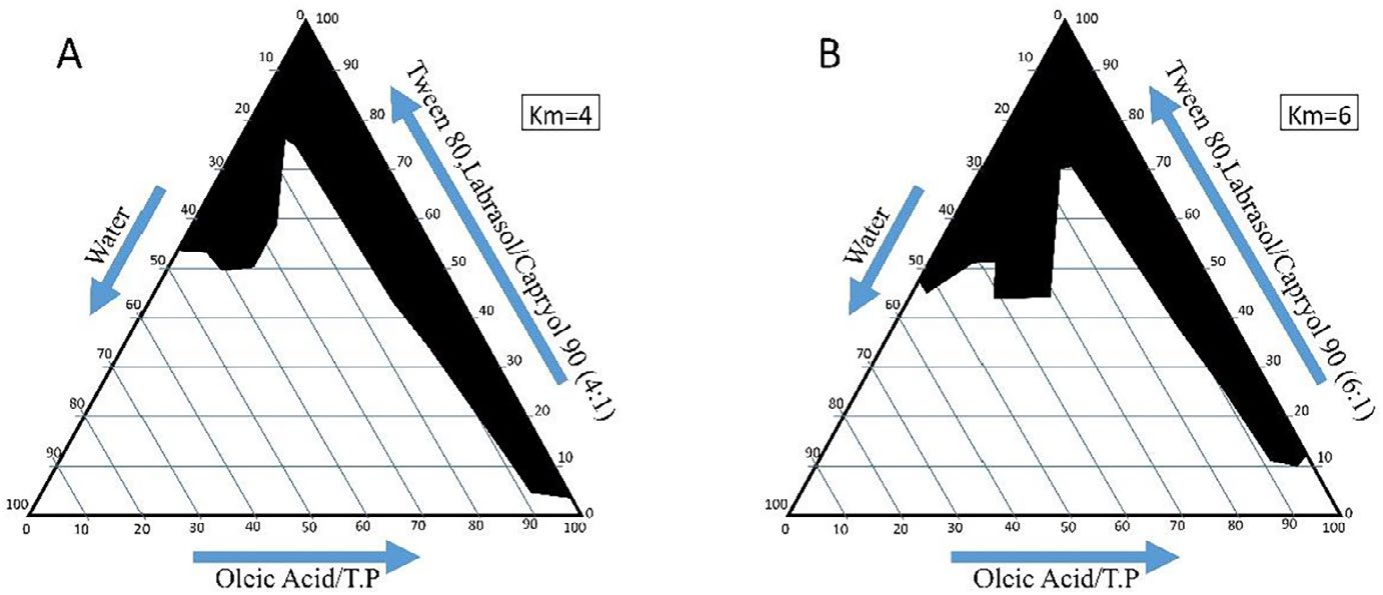
Pseudoternary phase diagrams of oil–surfactant/cosurfactant–water systems: A) 4:1 weight ratio of Labrasol‐Tween 80/Capryol 90 and B) 6:1 weight ratio of Labrasol‐Tween 80/Capryol 90. Dark areas indicate microemulsion zones.

To determine the ME zone for different S/C ratios, two‐phase diagrams were created (Figure 2). The S/C weight ratio significantly influences the phase behavior of the ME. Higher surfactant concentrations expanded the ME region. The phase diagrams clearly showed that the ME existence region grew as the S/C weight ratio increased, allowing more water to be incorporated into the ME structure (km = 4–6) [24].

### 3.3 Properties of VPA MEs

The viscosity, mean droplet size, and pH of the VPA MEs are shown in Table 3. The mean droplet size of the MEs ranged from 10 to 24 nm (Table 2). ME‐4 had the smallest particle size (10.25 ± 0.35 nm). Statistical analysis revealed no significant correlation between droplet size and the independent variables. However, a significant direct correlation was found between the viscosity of MEs and both the %Oil and S/C ratio. Conversely, the correlation between viscosity and %W was significant and inverse. The pH values of the MEs were compatible with skin (Table 3).

**TABLE 3:** Physicochemical properties of VPA microemulsions.

Formulation	pH	Mean Droplet Size (nm)	Polydispersity index	Viscosity (cps)
ME‐1	4.74 **±** 0.007	20.8 ± 1.41	0.334 ± 0.003	81.65 ± 0.49
ME‐2	5.12 **±** 0.0282	22.4 ± 1.97	0.336 ± 0.004	85.3 ± 0.98
ME‐3	4.88 **±** 0.0141	24.7 ± 1.72	0.332 ± 0.001	77.75 ± 1.06
ME‐4	5.225 **±** 0.021	10.25 ± 0.35	0.333 ± 0.003	84.15 ± 1.20
ME‐5	4.67 **±** 0.0141	19.35 ± 5.30	0.335 ± 0.002	79 ± 1.41
ME‐6	4.94 **±** 0.0141	24.2 ± 2.1	0.337 ± 0.004	78.5 ± 0.70
ME‐7	4.885 **±** 0.007	21.3 ± 1.69	0.336 ± 0.002	80.25 ± 1.06
ME‐8	4.605 **±** 0.007	24.3 ± 0.84	0.335 ± 0.001	80.05 ± 0.63

### 3.4 VPA Release Profiles

**FIGURE 3:**
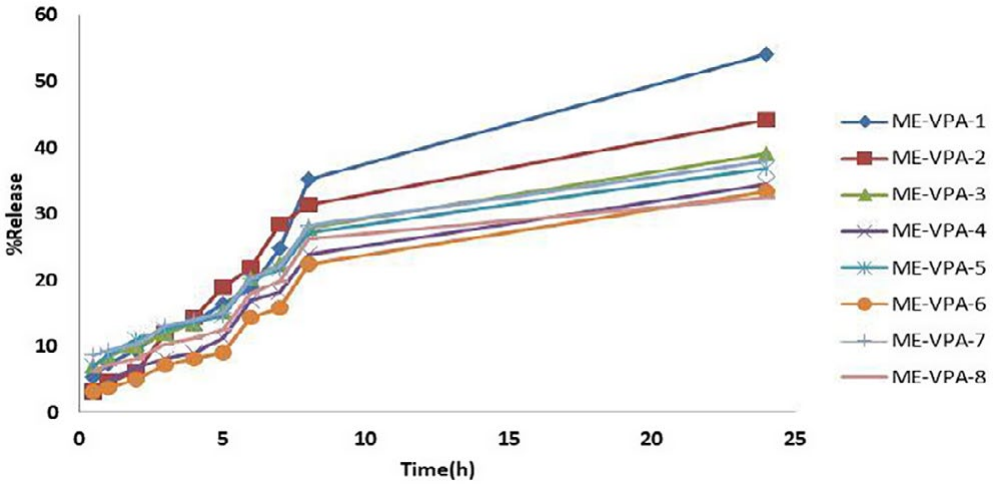
In vitro release profiles of VPA microemulsions (Mean ± SD, *n* = 3).

The percentage of released drug is a key feature of the formulation, significantly affecting therapeutic effectiveness. Figure 3 illustrates the release profiles of the VPA‐loaded MEs. These profiles indicate that 32%–54% of the drug was released over the 24‐h experimental period. This release pattern suggests a controlled release profile, which is beneficial for creating an effective drug depot in the skin. Table 4 presents the release percentages and kinetics of the VPA‐loaded MEs.

**TABLE 4:** Release kinetic models for VPA microemulsions.

Formulation	ME‐VPA‐1	ME‐VPA‐2	ME‐VPA‐3	ME‐VPA‐4	ME‐VPA‐5	ME‐VPA‐6	ME‐VPA‐7	ME‐VPA‐8
Drug release percentage (%Q_24_)	54.1542	44.2008	39.1791	34.5707	36.9198	33.4295	38.0972	32.4932
Kinetic	First	Higuchi	Higuchi	Higuchi	Higuchi	Higuchi	Higuchi	Higuchi
*R* ^2^	0.9517	0.9376	0.9461	0.94	0.9431	0.9326	0.9297	0.897
Slope	−0.0317	0.1086	0.0829	0.0805	0.0762	0.0785	0.0769	0.0704
Intercept	−0.0485	−0.0526	−0.0056	−0.0394	0.0065	−0.0494	0.0108	−0.0020

### 3.5 Physical Stability of MEs

The ME formulations remained physically stable for 6 months. No phase separation, flocculation, or coalescence occurred during storage at various temperatures or centrifugation.

### 3.6 In Vitro Permeation Studies Through Guinea Pig Skin

Hairy abdominal skin and nonhairy ear skin from guinea pigs served as models for epidermal and follicular pathways, respectively [19]. This study examined permeation through both skin types.

Permeability parameters of VPA‐loaded MEs were lower through hairy abdominal skin compared with nonhairy ear skin (Tables 5 and 6). The percentage of drug permeated after 8 and 24 h is shown in Table 7. Multivariate regression analysis revealed no significant correlation (*p* > 0.05) between independent variables and the following permeability parameters: *T*_lag_, *D*_app_, nonhairy ear skin *J*_ss_, and nonhairy ear skin *P*. However, a significant correlation (*p* < 0.05) emerged between independent variables and both hairy abdominal skin *J*_ss_ and *P* parameters. Specifically, the S/C percentage in ME formulations significantly influenced these parameters, with higher S/C percentages leading to increased *J*_ss_ and *P* values in hairy abdominal skin.

**TABLE 5:** In vitro permeability parameters of VPA microemulsions through guinea pig abdominal skin (mean ± SD, *n* = 3).

Formulation	*J*_ss_ (mg/cm^2^ h)	*P* (cm/h)	*D* (cm^2^/h)	*T*_lag_ (h)	ER_flux_	ER_D_
Control	0.133 ± 0.013	1.61 ± 0.16	0.118 ± 0.003	0.9 ± 0.02	—	—
ME‐VPA‐1	0.793 **±** 0.006	9.56 **±** 0.08	0.223 ± 0.013	0.48 ± 0.03	5.97 ± 0.55	1.89 ± 0.15
ME‐VPA‐2	0.743 **±** 0.033	8.96 **±** 0.41	0.38 ± 0.29	0.4 ± 0.32	5.58 ± 0.3	3.17 ± 2.42
ME‐VPA‐3	0.865 **±** 0.011	10.42 **±** 0.14	0.045 ± 0.001	2.39 ± 0.07	6.52 ± 0.74	0.38 ± 0.02
ME‐VPA‐4	1.177 **±** 0.029	14.18 **±** 0.35	0.116 ± 0.008	0.92 ± 0.07	8.88 ± 1.1	0.98 ± 0.05
ME‐VPA‐5	0.34 **±** 0.02	4.093 **±** 0.24	0.065 ± 0.01	1.67 ± 0.24	2.55 ± 0.1	0.55 ± 0.06
ME‐VPA‐6	0.53 **±** 0.004	6.44 **±** 0.058	0.26 ± 0.02	0.42 ± 0.03	4.02 ± 0.44	2.17 ± 0.1
ME‐VPA‐7	0.795 ± 0.011	9.58 ± 0.14	0.075 ± 0.003	1.42 ± 0.065	5.99 ± 0.68	0.64 ± 0.01
ME‐VPA‐8	0.36 ± 0.020	4.34 ± 0.24	0.07 ± 0.004	1.48 ± 0.09	2.7 ± 0.12	0.61 ± 0.05

**TABLE 6:** In vitro permeability parameters of VPA microemulsions through guinea pig ear skin (mean ± SD, *n* = 3).

Formulation	*J*_ss_ (mg/cm^2^ h)	*P* (cm/h)	D (cm^2^/h)	$T_{\mathrm{lag}}$ (h)	ERflux	ERD
Control	0.44 ± 0.02	5.29 ± 0.23	0.19 ± 0.001	0.22 ± 0.001	—	—
ME‐VPA‐1	0.92 ± 0.017	11.05 ± 0.21	0.03 ± 0.001	1.44 ± 0.04	2.09 ± 0.05	0.15 ± 0.004
ME‐VPA‐2	1.86 ± 0.021	22.36 ± 0/25	0.59 ± 0.16	0.07 ± 0.02	4.23 ± 0.23	3.11 ± 0.82
ME‐VPA‐3	1.91 ± 0.002	23.004 ± 0.03	0.28 ± 0.003	0.15 ± 0.001	4.35 ± 0.2	1.49 ± 0.01
ME‐VPA‐4	1.28 ± 0.135	15.38 ± 1.63	0.24 ± 0.27	0.44 ± 0.49	2.91 ± 0.43	1.29 ± 1.42
ME‐VPA‐5	1.06 ± 0.017	12.73 ± 0.21	0.17 ± 0.046	0.26 ± 0.072	2.4 ± 0.146	0.89 ± 0.24
ME‐VPA‐6	2.12 ± 0.005	25.61 ± 0.06	0.02 ± 0.001	1.94 ± 0.01	4.84 ± 0.2	0.11 ± 0.001
ME‐VPA‐7	2.09 ± 0.017	25.18 ± 0.21	0.02 ± 0.001	1.89 ± 0.04	4.76 ± 0.25	0.11 ± 0.002
ME‐VPA‐8	1.17 ± 0.052	14.10 ± 0.63	0.17 ± 0.1	0.3 ± 0.19	2.67 ± 0.23	0.89 ± 0.24

**TABLE 7:** Percentage of VPA permeated through hairy and nonhairy guinea pig skin (mean ± SD, *n* = 3).

Formulation	Hairy abdominal skin		Nonhairy ear skin	
	Drug% 8 h	Drug% 24 h	Drug% 8 h	Drug% 24 h
Control	2.008 ± 0.012	2.441 ± 0.032	3.179 ± 0.026	3.707 ± 0.029
ME‐VPA‐1	12.647 ± 0.06	15.84 ± 0.044	11.850 ± 0.048	14.85 ± 0.107
ME‐VPA‐2	12.29 ± 0.079	15.295 ± 0.03	13.514 ± 0.110	16.51 ± 0.107
ME‐VPA‐3	17.492 ± 0.033	20.46 ± 0.053	14.997 ± 0.065	18.437 ± 0.169
ME‐VPA‐4	13.416 ± 0.036	16.322 ± 0.115	11.365 ± 0.074	14.036 ± 0.146
ME‐VPA‐5	8.895 ± 0.063	9.91 ± 0.028	7.284 ± 0.071	8.555 ± 0.084
ME‐VPA‐6	12.811 ± 0.117	15.041 ± 0.085	10.825 ± 0.058	12.819 ± 0.097
ME‐VPA‐7	8.861 ± 0.054	12.049 ± 0.096	10.693 ± 0.029	14.305 ± 0.065
ME‐VPA‐8	5.243 ± 0.043	7.152 ± 0.028	9.039 ± 0.078	11.714 ± 0.045

## 4 Discussion

VPA, a histone deacetylase inhibitor, is commonly used to treat various forms of epilepsy, bipolar disorder, and migraine prophylaxis, with additional off‐label applications. While oral VPA can cause hair loss, research on its topical form has shown potential for hair growth. This study aimed to examine the follicular contribution to VPA ME skin passage and investigate how the carrier affects the drug's skin kinetics [2, 3].

Developing and studying different MEs for transdermal delivery is valuable, as the relationship between ME composition and drug delivery efficacy is well‐established. To explore follicular contribution to skin passage, enhance drug penetration, and examine carrier effects on skin kinetics, we formulated a new VPA preparation. We compared transfollicular and skin permeability rates using pig ear skin (transdermal) and abdominal skin (transfollicular).

Our ME analysis revealed that VPA solubility in oleic acid alone as an oil phase is 78 mg/mL. However, combining transcotol P with oleic acid (1:10 ratio) significantly increased VPA solubility to 81.5 mg/mL. This aligns with Salimi et al.'s 2013 study on celecoxib delivery through rat skin, where transcotol P with oleic acid increased celecoxib solubility from 2.1 to 6.49 mg/mL [25]. Similarly, Salimi et al.'s 2017 study on azithromycin ocular delivery in rabbit corneas showed increased solubility from 5.1 to 9.3 mg/mL with this combination [26]. Boelsma et al.'s [27] research supports the use of oleic acid in formulations, confirming its nonirritating properties.

The VPA ME particle sizes ranged from 10.25 to 24.7 nm, falling within the ME range of less than 100 nm. Smaller particles generally penetrate the skin more easily. Patro et al.'s 2010 study on VPA ME observed particle sizes between 0.2 and 7 nm [28]. Our research found no significant relationship between particle size and independent variables.

Formulation viscosity is crucial for ME persistence on the skin. Our viscosity analysis showed formulations 2 and 3 had the highest (85.3) and lowest (77.75) viscosities at 50 rpm, respectively. Kawtikwar et al.'s 2009 study on VPA ME reported viscosities between 27.5 and 46.5 cps [29]. We found significant relationships between ME viscosity and surfactant‐to‐cosurfactant ratio, oil percentage, and water percentage. Higher oil percentages and surfactant‐to‐cosurfactant ratios, along with lower water percentages, increased ME viscosity.

Given VPA's topical application in this study, the formulation's pH is crucial for skin compatibility. ME pH values ranged from 4.65 to 5.22. Analysis of variance revealed a significant relationship between pH and both the surfactant‐to‐cosurfactant ratio and water percentage. Lower water percentages and higher surfactant‐to‐cosurfactant ratios increased ME pH. The observed pH range aligns well with the ideal 4.5–6.5 range for skin products [30].

Drug release studies showed formulations 1 (30% water, 10% oil, 60% (S + C) at 6:1 ratio) and 8 (30% water, 10% oil, 60% (S + C) at 4:1 ratio) had the highest (54.15%) and lowest (32.49%) release percentages, respectively. This contrasts with Kawtikwar et al.'s 2009 study on VPA ME, which reported release rates between 12.45% and 13.49% after 3 h [29]. Formulation 1 followed a first‐order release model, while others followed the Higuchi model. Analysis of variance showed no significant relationship between particle size and drug release percentage after 8 or 24 h.

However, the 24‐h drug release percentage correlated significantly with the oil percentage and surfactant‐to‐cosurfactant ratio. Higher oil percentages and surfactant‐to‐cosurfactant ratios increased 24‐h release in MEs. The 8‐h release rate showed a significant relationship with oil percentage, with higher oil percentages increasing release rates.

We also examined ME formulations' effects on VPA permeability parameters in both abdominal (with hair) and ear (without hair) pig skin. Compared with the water saturation control (3.8% VPA solution), ME effects were assessed using *J*_ss_, *P*, *D*, *T*_lag_ indices, and drug passage percentages after 8 and 24 h.

In the first four formulations (6:1 surfactant‐to‐cosurfactant ratio), 24‐h drug passage through the abdominal route exceeded that of the ear route, indicating significant follicular passage. The second four formulations (4:1 ratio) showed higher 24‐h passage through the ear route, suggesting predominant epidermal passage. Most formulations accelerated drug passage through the follicular pathway.

The surfactant‐to‐cosurfactant ratio significantly influenced *J*_ss_ in abdominal skin, with higher ratios increasing *J*_ss_. This aligns with Sharif Makhmalzadeh et al.'s 2012 study on optimizing ibuprofen skin delivery [31]. Tabosa et al. noted that high *J*_ss_ can create higher drug concentrations in upper skin layers and enhance penetration into blood flow [32, 33].

Tlag showed no significant relationship with independent variables in either skin type. The permeability coefficient (*P*) in abdominal skin correlated significantly with the surfactant‐to‐cosurfactant ratio, increasing as the ratio rose. *P* in ear skin showed no significant relationships with studied parameters.

The parameter *D* for both abdominal and ear skin regions showed no significant relationship with any of the independent variables studied.

## 5 Conclusion

This study demonstrates the potential microemulsions formulations for enhancing the topical delivery of VPA, a histone deacetylase inhibitor with therapeutic applications beyond its common use in seizure disorders. Our key findings include:

1. ME formulations significantly improved VPA penetration through both epidermal and follicular pathways compared to aqueous solutions.
2. The composition of MEs, particularly the water content, oil content, and surfactant‐to‐cosurfactant (S/C) ratio, crucially influenced their physicochemical properties and skin permeation parameters.
3. Higher S/C ratios in ME formulations correlated with increased steady‐state flux (*J*_ss_) and permeability coefficient (*P*) in hairy skin, suggesting enhanced follicular delivery.
4. ME formulations exhibited controlled release profiles, with 32%–54% of VPA released over 24 h, potentially creating an effective drug depot in the skin.

These findings provide valuable insights for developing topical VPA formulations with improved skin penetration and follicular delivery. Such formulations could offer new therapeutic approaches for conditions requiring targeted VPA delivery to the skin or hair follicles, such as certain dermatological disorders or hair regeneration therapies.

Future research should focus on optimizing ME compositions for specific therapeutic applications, evaluating their long‐term stability, and conducting in vivo studies to assess their efficacy and safety in relevant disease models.

## Author Contributions

A.S. conceived and designed the evaluation and drafted the manuscript. S.M.S. participated in designing the evaluation, performed parts of the statistical analysis, and helped to draft the manuscript. A.S., Ar.S., and S.M.S. re‐evaluated the data, revised the manuscript, and performed the statistical analysis, and revised the manuscript. S.J. collected the clinical data, interpreted them, and revised the manuscript. Final approval of the version to be published: A.S., S.J., Ar.S., and S.M.S.

## Ethics Statement

The Ethics Committee of Jundishapur University of Medical Sciences (IR.AJUMS.ABHC.REC.1399.004) on 03.03.2020 provided ethics approval for this study.

## Conflicts of Interest

The authors declare no conflicts of interest.

## Declarations

This paper has been approved by all coauthors and the responsible authorities at the institute where the research has been carried out.

## Data Availability

The data that support the findings of this study are available from the corresponding author upon reasonable request.
